# Fusion-specific co-mutational architecture and treatment-response associations in pediatric core-binding factor acute myeloid leukemia: a multicenter exome-profiled cohort

**DOI:** 10.3389/fonc.2026.1897872

**Published:** 2026-09-30

**Authors:** Mei Li, Chunxia Cai, Chunping Wu, Xiaoqin Feng, Mincui Zheng, Huirong Mai, Lihua Yang, Xiangling He, Hong Wen, Shaohua Le, Nainong Li, Yongzhi Zheng

**Affiliations:** 1 Department of Pediatric Hematology, Fujian Institute of Hematology, Fujian Provincial Key Laboratory on Hematology, Fujian Medical University Union Hospital, Fuzhou, China; 2 Department of Pediatrics, Nanfang Hospital, Southern Medical University, Guangzhou, China; 3 Department of Pediatric Hematology/Oncology, Hematology and Oncology, Hunan Children’s Hospital, Changsha, China; 4 Department of Pediatric Hematology/Oncology, Shenzhen Children’s Hospital, Shenzhen, China; 5 Department of Pediatrics, Zhujiang Hospital of Southern Medical University, Guangzhou, China; 6 Department of Pediatrics, Paediatrics, People’s Hospital of Hunan Province, Changsha, China; 7 Paediatrics, The First Affiliated Hospital of Xiamen University, Xiamen, China

**Keywords:** CBFB::MYH11, co-mutation, core-binding factor, measurable residual disease, pediatric acute myeloid leukemia, RUNX1::RUNX1T1, whole-exome sequencing

## Abstract

**Introduction:**

Core-binding factor acute myeloid leukemia (CBF-AML) is defined by one of two founding fusions, but cooperating lesions may differ between fusion subtypes. We characterized the broader diagnostic co-mutational architecture of pediatric CBF-AML and examined its relationship with post-induction measurable residual disease (MRD) and outcome.

**Methods:**

This retrospective analysis included 170 children with diagnostic whole-exome sequencing (WES) data (2019–2023), a subset of a reported 289-patient cohort. Candidate leukemia-associated variants were curated conservatively because matched normal samples were unavailable. Variant frequencies were compared between RUNX1::RUNX1T1 and CBFB::MYH11 AML with false-discovery-rate control. Associations with MRD were tested using Fisher’s exact test. Primary feature-specific Cox models for event-free survival (EFS) were adjusted only for fusion subtype and induction regimen; sensitivity analyses additionally addressed age and presenting WBC count, MRD, and transplantation.

**Results:**

In a limited selection audit, fusion-subtype and FLT3-ITD distributions were similar between WES-included and non-included patients, whereas clinical-source KIT positivity was more frequent in the WES subset. The analytic cohort included 138 RUNX1::RUNX1T1 and 32 CBFB::MYH11 cases; 137 received FLAG-IDA, and 33 received DAE induction. At least one curated candidate variant was present in 135 patients (79.4%), and 69 (40.6%) had alterations in two or more genes. Tyrosine-kinase signaling alterations occurred in 115 patients (67.6%). RAS-pathway alterations were enriched in CBFB::MYH11 AML (53.1% vs. 19.6%; P < 0.001, q=0.002), driven principally by NRAS (50.0% vs. 15.2%; P < 0.001, q < 0.001). Chromatin-modifier alterations were numerically enriched in RUNX1::RUNX1T1 AML (24.6% vs. 6.2%; P = 0.028) but did not remain significant after multiplicity correction (q=0.114). After false-discovery-rate correction, no tested genomic feature was associated with MFC-MRD or molecular MRD positivity after the second induction. With a median follow-up of 33.2 months, 3-year EFS and overall survival were 84.2% and 89.8%, respectively. No baseline genomic feature showed a significant association with EFS in either the primary or expanded baseline-covariate models.

**Conclusions:**

Among WES-profiled patients, pediatric CBF-AML showed fusion-specific co-mutational architecture, especially RAS-pathway enrichment in CBFB::MYH11 AML. In this WES-defined cohort, baseline genomic features were not established as response or outcome predictors. These findings are descriptive and hypothesis-generating; they do not support treatment-specific causal inference.

## 1 Introduction

Core-binding factor acute myeloid leukemia (CBF-AML) is defined by RUNX1::RUNX1T1 or CBFB::MYH11 and is recognized as a distinct disease family in contemporary classifications (1, 2). Both entities are generally assigned to favorable-risk categories, yet favorable risk is not synonymous with uniform cure: relapse and treatment-related mortality remain clinically important, particularly in children whose disease biology and treatment exposures differ from those of adults (3, 4). The founding fusions disrupt the core-binding factor transcriptional complex and hematopoietic differentiation, but are usually insufficient for leukemogenesis without additional cooperating lesions (5).

Large-scale genomic studies have established that AML develops through combinations of lesions affecting signaling, chromatin regulation, transcription, cohesin, and RNA splicing (6, 7). Pediatric AML is not simply a younger version of adult AML; it contains age-dependent fusion and mutational spectra, a lower burden of age-related epigenetic lesions, and distinct structural alterations (8). A national Chinese pediatric series likewise demonstrated population- and age-relevant genomic patterns that can alter conventional risk classification (9). These observations underscore the need for pediatric, treatment-contextualized analyses rather than assuming that adult effect estimates are directly transferable.

CBF-AML-specific sequencing studies have repeatedly shown a high prevalence of receptor-tyrosine-kinase and RAS-pathway lesions, with fusion-dependent organization of the cooperating genome (10–13). NRAS and related RAS-pathway alterations are especially frequent in CBFB::MYH11 AML (14), whereas ASXL2 and other chromatin-regulatory alterations are concentrated in RUNX1::RUNX1T1 AML and can cooperate experimentally with the fusion (15). Pediatric studies confirm frequent KIT and RAS lesions but have reported inconsistent mutation-outcome associations across cooperative groups, fusion subtypes, allelic classes, treatment protocols, and study eras (16–19). Clonal competition, mutation burden, and molecular response may be more informative than binary mutation status alone (20, 21), and pediatric fusion-specific outcome series underscore the danger of pooling biologically different CBF entities (22).

Our group recently reported the associations of KIT, FLT3-ITD, post-induction measurable residual disease (MRD), and induction regimen with outcomes in 289 children treated on the C-HUANAN-AML-15 protocol (23). Every patient in the present 170-patient exome-profiled cohort was included in that earlier clinical cohort; therefore, this study is not an independent validation of those findings. We instead asked a distinct question: how the broader, conservatively curated co-mutational architecture differs between the two CBF fusion subtypes, and whether prespecified diagnostic genomic features are associated with post-induction MRD or subsequent outcome. KIT and FLT3 are retained as elements of the landscape, rather than as the primary focus of the study.

## 2 Materials and methods

### 2.1 Study design, cohort selection, and relationship to the prior publication

This seven-center retrospective cohort comprised 170 consecutive children aged <14 years who were diagnosed with *de novo* CBF-AML in South China from January 2019 through December 2023 and had diagnostic whole-exome sequencing (WES) data available in the analytic dataset. CBF-AML was defined by RUNX1::RUNX1T1 or CBFB::MYH11 in accordance with contemporary disease classifications (1, 2). Therapy-related AML and Down syndrome-associated myeloid leukemia were excluded. WES availability, rather than induction regimen, defined entry into this analysis.

All 170 participants were included in the previously published 289-patient C-HUANAN-AML-15 cohort, representing 100% of the present cohort and 58.8% of the earlier cohort (23). The earlier report included diagnoses from 2015 through 2023 and evaluated selected mutations, MRD, induction regimen, and survival in the full clinical cohort. The present WES-defined analysis focuses on broader gene- and pathway-level architecture, fusion-specific differences, and exploratory associations of diagnostic genomic features with treatment response and outcome. No patient is presented as an independent validation case for a result reported previously.

To assess selection into the WES-defined cohort, we performed a *post hoc* comparison of the 170 included patients with the 119 patients from the previously reported CBF-AML cohort who were not included. The comparison was restricted to characteristics recoverable under harmonized definitions from the current analytic file and published full-cohort counts: fusion subtype, clinically ascertained KIT positivity, and FLT3-ITD. Counts for the non-included group were obtained by subtracting the WES-subset counts from the 289-patient totals. The clinical-source KIT definition used for this audit reflects the earlier diagnostic workflow (PCR before 2019 and WES thereafter) and is not identical to the conservative candidate-variant definition used in the current genomic matrix. Comparisons used Fisher’s exact test and absolute standardized differences and are descriptive (**Supplementary Table S10**).

### 2.2 Treatment and response assessment

Patients received the nonrandomized C-HUANAN-AML-15 protocol (24), summarized in **Supplementary Figure S1**. Induction comprised either two tandem FLAG-IDA courses (fludarabine, cytarabine, granulocyte colony-stimulating factor, and idarubicin) or two DAE courses (daunorubicin, cytarabine, and etoposide). Consolidation was cytarabine-based and included homoharringtonine, cytarabine, and etoposide (HAE) or homoharringtonine and high-dose cytarabine (HHA), followed by mitoxantrone and cytarabine (MidAC), according to the assigned treatment branch. Allocation reflected physician and institutional practice. Allogeneic hematopoietic stem-cell transplantation (HSCT) in first complete remission (CR1) was used selectively according to response and clinical risk. No patient with an FLT3 alteration received an FLT3 inhibitor during frontline or post-remission therapy.

MRD was assessed after induction course 1 (TP1) and after induction course 2 before consolidation (TP2). Multiparameter flow cytometry MRD (MFC-MRD) combined leukemia-associated immunophenotype and different-from-normal approaches; positivity was defined as ≥0.1%, as specified in the protocol (24). Fusion-transcript molecular MRD was measured by quantitative reverse-transcription PCR relative to ABL1 and analyzed using the protocol-recorded binary result. Although MRD was recorded at two protocol time points, the available variables were binary and were not modeled as continuous serial trajectories or log-reduction kinetics. The clinical interpretation of post-induction MRD was informed by pediatric cooperative-group data and current consensus recommendations (25, 26).

### 2.3 Whole-exome sequencing and conservative variant curation

DNA from diagnostic peripheral blood leukocytes underwent library preparation and exome capture with Twist reagents, followed by paired-end sequencing on an Illumina NovaSeq X Plus platform. Reads were aligned to hg19/GRCh37 with BWA-MEM, an implementation of the Burrows–Wheeler alignment algorithm (27), and annotated with the Ensembl Variant Effect Predictor (28). Common variants with minor-allele frequency >1% in the 1000 Genomes Project (29), gnomAD (30), or the institutional database were filtered. Protein-altering and splice-region variants were prioritized and reviewed using available pathogenicity predictions, ClinVar (31), and the Human Gene Mutation Database (32), with interpretive principles informed by American College of Medical Genetics and Genomics/Association for Molecular Pathology guidance (33).

Matched remission or other germline DNA was unavailable. Because tumor-only sequencing cannot reliably distinguish every somatic event from an unrecognized germline variant (34), we use the term candidate leukemia-associated variant rather than confirmed somatic mutation, and no constitutional or germline inference is made from these data. A KIT p.M541L call explicitly labeled germline in the source dataset was excluded; two patients who also carried KIT p.N822K remained KIT-positive on the basis of p.N822K. The source WT1 flag mixed sequence variants with elevated expression or transcript annotations, so all 15 WT1-flagged records were excluded from the primary matrix. Original variant-call files and standardized coverage/depth metrics were unavailable; WES sensitivity could not be benchmarked against a deep targeted assay, and low-allele-fraction, copy-number, or structural events may have been missed. The complete primary-analysis rules for somatic-status terminology, population filtering, KIT p.M541L, WT1, FLT3 classification, alteration counting, pathway coding, and co-occurrence eligibility are summarized in **Supplementary Table S1**.

### 2.4 Functional grouping and genomic features

Genes were assigned *a priori* to functional categories based on established CBF-AML genomic and clonal studies (10, 11, 20): tyrosine-kinase signaling (KIT, FLT3, NRAS, KRAS, JAK2, PTPN11, CBL, and CSF3R); RAS (NRAS and KRAS); receptor tyrosine kinase (RTK; KIT and FLT3); chromatin modifiers (ASXL1, ASXL2, KDM6A, EZH2, BCORL1, and KMT2A); DNA methylation (TET2, DNMT3A, and IDH2); cohesin (RAD21, SMC1A, SMC3, and STAG2); transcription factors (ETV6, RUNX1, and IKZF1); and spliceosome (SRSF2). A patient was positive for a category if at least one member gene was altered. Alteration count denotes the number of altered curated genes, not individual variant calls. The prespecified membership of each functional category is listed in **Supplementary Table S2**.

### 2.5 Outcome definitions

Complete remission was defined as <5% bone marrow blasts with hematologic recovery. Event-free survival (EFS) was measured from diagnosis to the earliest database-recorded event, with censoring at the last follow-up. Induction failure was prespecified as failure to achieve complete remission after two induction courses; no patient met this definition, so no induction-failure date was assigned or imputed. Observed EFS events were relapse, death, or protocol discontinuation due to transfer of care, treatment change for intolerance, or treatment abandonment. Overall survival (OS) was measured from diagnosis to death from any cause. Cumulative incidence of relapse (CIR) was measured from complete remission to relapse, treating death without relapse as a competing event.

### 2.6 Statistical analysis

Continuous variables were summarized as median and interquartile range and compared with the Mann–Whitney U test; categorical variables were compared with Fisher’s exact test. To limit false-positive findings in the setting of many genomic comparisons, Benjamini–Hochberg false-discovery-rate control was applied separately within prespecified families of fusion comparisons, MRD comparisons, survival models, and pairwise co-occurrence tests (35). Both nominal P values and adjusted q values are reported; q < 0.05 defined statistical significance after multiplicity correction.

EFS and OS were estimated by the Kaplan–Meier method (36), with median follow-up calculated by reverse Kaplan–Meier estimation (37). CIR was estimated with an Aalen–Johansen transition-probability approach (38), appropriate for relapse in the presence of the competing risk of nonrelapse death (39). Primary feature-specific Cox proportional hazards models (40) evaluated one prespecified genomic feature at a time while adjusting only for fusion subtype and induction regimen. No stepwise selection, multifeature genomic model, interaction term, or genomic-feature-by-treatment analysis was fitted. To assess residual baseline confounding, a separate complete-case sensitivity analysis additionally included age at diagnosis (continuous, years) and presenting WBC count [log-transformed as log(1+WBC), with WBC measured in ×10^9^/L] in each feature-specific model. Because only 25 EFS events and 14 deaths occurred, the primary models were intentionally parsimonious, and the expanded models were considered exploratory; neither analysis was interpreted as establishing full independence from all baseline prognostic factors.

TP2 MFC-MRD is post-baseline and may mediate, rather than confound, an association between diagnostic genomics and EFS; it was therefore added only in a secondary complete-case sensitivity model. Eventual CR1-HSCT was not entered as a fixed baseline covariate because transplant dates were unavailable and such coding would risk immortal-time bias. Instead, EFS models were repeated after excluding CR1-HSCT recipients. These analyses were not designed to estimate treatment effects or mutation-specific treatment benefit. Analyses were performed with Python 3.12, pandas, SciPy, and prespecified custom survival routines. All tests were two-sided.

### 2.7 Ethical considerations

The study was approved by the Ethics Committee of Fujian Medical University Union Hospital (approval number 2025KY666). The requirement for individual informed consent was waived for this retrospective analysis in accordance with institutional requirements and the Declaration of Helsinki.

## 3 Results

### 3.1 Cohort characteristics and selection audit

The analysis included 138 patients (81.2%) with RUNX1::RUNX1T1 and 32 (18.8%) with CBFB::MYH11. Median age was 103.0 months (interquartile range [IQR], 67.2–139.0), and 103 patients (60.6%) were male. Thirty-four of 168 evaluable patients (20.2%) had a presenting white blood cell count ≥50 × 10^9^/L. FLAG-IDA was used in 137 patients (80.6%) and DAE in 33 (19.4%) as the first induction course; 64 patients (37.6%) underwent allogeneic HSCT in CR1 (**Supplementary Figure S2**). Patients with CBFB::MYH11 AML were older at diagnosis than those with RUNX1::RUNX1T1 AML (median age, 130.0 vs 92.0 months; P = 0.022) and presented with a higher median white blood cell count (73.8 vs 17.1 × 10^9^/L; P < 0.001). Induction regimen, CR1-HSCT, and recorded MRD positivity did not differ significantly by fusion subtype (**Table 1**).

**Table 1:** Clinical characteristics by CBF fusion subtype.

Characteristic	Overall	RUNX1::RUNX1T1	CBFB::MYH11	P
Age, months	103.0 (67.2-139.0)	92.0 (65.8-131.2)	130.0 (93.0-147.0)	0.022
WBC, x10^9/L	18.9 (9.3-39.2)	17.1 (8.6-30.4)	73.8 (16.8-180.9)	<0.001
Male sex	103/170 (60.6%)	80/138 (58.0%)	23/32 (71.9%)	0.165
WBC >= 50 x10^9/L	34/168 (20.2%)	15/136 (11.0%)	19/32 (59.4%)	<0.001
Extramedullary disease	35/170 (20.6%)	28/138 (20.3%)	7/32 (21.9%)	0.812
FLAG-IDA induction	137/170 (80.6%)	110/138 (79.7%)	27/32 (84.4%)	0.629
Allogeneic HSCT in CR1	64/170 (37.6%)	54/138 (39.1%)	10/32 (31.2%)	0.544
MFC-MRD positive after induction 1	58/162 (35.8%)	50/131 (38.2%)	8/31 (25.8%)	0.218
MFC-MRD positive after induction 2	29/156 (18.6%)	25/126 (19.8%)	4/30 (13.3%)	0.602
Molecular MRD positive after induction 2	77/156 (49.4%)	64/126 (50.8%)	13/30 (43.3%)	0.544

Values are median (interquartile range) or n/N (%). Denominators reflect available data. CBF, core-binding factor; CR1, first complete remission; HSCT, hematopoietic stem-cell transplantation; MFC, multiparameter flow cytometry; MRD, measurable residual disease; WBC, white blood cell count.

In the limited selection audit, the proportion with RUNX1::RUNX1T1 was similar in WES-included and non-included patients (81.2% vs 78.2%; absolute standardized difference, 0.075; Fisher’s exact P = 0.553), as was FLT3-ITD positivity (4.7% vs 6.7%; absolute standardized difference, 0.087; P = 0.602). In contrast, KIT positivity under the prior clinical-source definition was more frequent in the WES subset (43.5% vs 24.4%; absolute standardized difference, 0.413; P = 0.001). Because the WES subset was restricted to seven centers and 2019–2023, and because the source-defined KIT variable spans different diagnostic workflows, this comparison cannot separate clinical selection from era-, center-, and assay-related ascertainment. It demonstrates that the WES subset should not be assumed to represent the complete 289-patient cohort (**Supplementary Table S10**).

### 3.2 Curated genomic landscape

After conservative curation, 135 patients (79.4%) carried at least one candidate leukemia-associated variant, comprising 239 patient–gene events. The median number of altered genes was 1, and 69 patients (40.6%) had alterations in at least two genes. Tyrosine-kinase signaling was the dominant functional category (115/170, 67.6%), followed by RTK alterations (86/170, 50.6%), RAS alterations (44/170, 25.9%), and chromatin-modifier alterations (36/170, 21.2%). The most frequently altered genes were KIT (73/170, 42.9%), NRAS (37/170, 21.8%), FLT3 (20/170, 11.8%), KRAS (15/170, 8.8%), ASXL2 (15/170, 8.8%), CSF3R (14/170, 8.2%), ASXL1 (12/170, 7.1%), and CBL (11/170, 6.5%) (**Figure 1**; **Table 2**). The curated variant-level distribution of KIT is provided in **Supplementary Table S3**.

**Figure 1:**
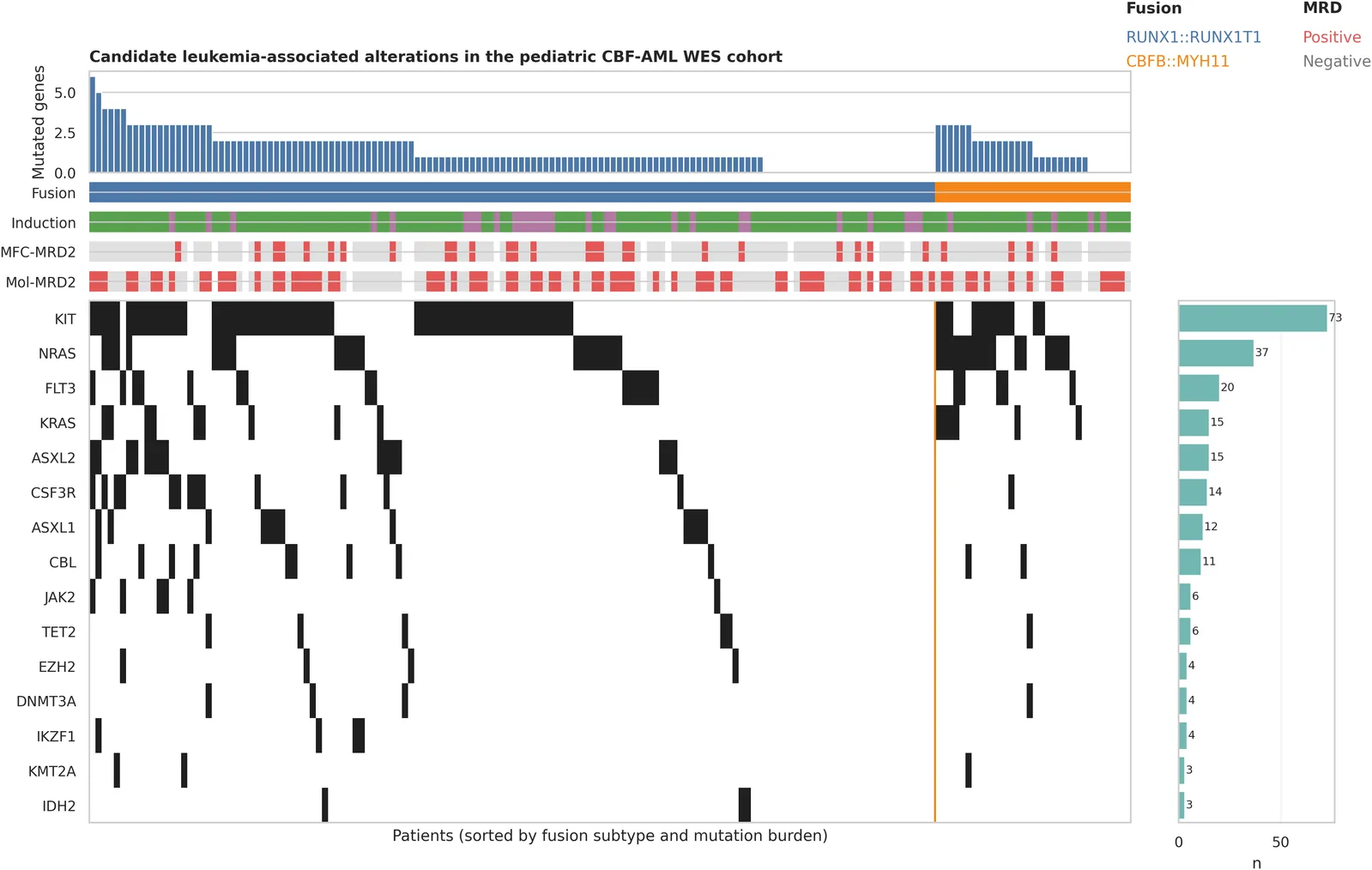
Curated candidate leukemia-associated variants in the pediatric CBF-AML WES cohort. Patients are ordered by fusion subtype and alteration count. The upper bars show the number of altered curated genes per patient; annotation tracks show fusion, induction regimen, TP2 MFC-MRD, and TP2 molecular MRD. The matrix includes genes altered in at least three patients. White cells indicate no recorded alteration. WT1 was excluded because the source flag mixed expression and sequence-variant records.

**Table 2:** Curated genomic features by CBF fusion subtype.

Feature	Overall	RUNX1::RUNX1T1	CBFB::MYH11	P	q
Any candidate leukemia-associated variant	135/170 (79.4%)	110/138 (79.7%)	25/32 (78.1%)	0.812	0.812
≥2 altered genes	69/170 (40.6%)	53/138 (38.4%)	16/32 (50.0%)	0.238	0.476
Tyrosine-kinase signaling	115/170 (67.6%)	92/138 (66.7%)	23/32 (71.9%)	0.677	0.697
RTK (KIT/FLT3)	86/170 (50.6%)	71/138 (51.4%)	15/32 (46.9%)	0.697	0.697
RAS	44/170 (25.9%)	27/138 (19.6%)	17/32 (53.1%)	<0.001	0.002
Chromatin modifiers	36/170 (21.2%)	34/138 (24.6%)	2/32 (6.2%)	0.028	0.114
DNA methylation	10/170 (5.9%)	9/138 (6.5%)	1/32 (3.1%)	0.690	0.697
Cohesin	5/170 (2.9%)	5/138 (3.6%)	0/32 (0.0%)	0.585	0.697
KIT	73/170 (42.9%)	61/138 (44.2%)	12/32 (37.5%)	0.555	0.860
NRAS	37/170 (21.8%)	21/138 (15.2%)	16/32 (50.0%)	<0.001	<0.001
KRAS	15/170 (8.8%)	9/138 (6.5%)	6/32 (18.8%)	0.039	0.256
FLT3 (any)	20/170 (11.8%)	15/138 (10.9%)	5/32 (15.6%)	0.541	0.860
CSF3R	14/170 (8.2%)	13/138 (9.4%)	1/32 (3.1%)	0.473	0.860
ASXL1	12/170 (7.1%)	12/138 (8.7%)	0/32 (0.0%)	0.126	0.409
ASXL2	15/170 (8.8%)	15/138 (10.9%)	0/32 (0.0%)	0.077	0.333

P values are from Fisher’s exact test. q values are Benjamini–Hochberg adjusted within summary, pathway, or gene/subtype families as specified in the analysis. WT1 was excluded from the primary matrix; a KIT p.M541L-only germline-labeled call was removed.

The verified FLT3 classification comprised eight ITD and 12 other non-ITD alterations (**Supplementary Table S4**). Because the source annotations were not sufficiently uniform for reliable subclassification, all non-ITD alterations—including records previously labeled as tyrosine-kinase-domain variants—were grouped as other FLT3 alterations and were not modeled separately. Pairwise testing among recurrently altered genes yielded nominal associations, but no co-occurrence pair remained significant after false-discovery-rate correction (all q≥0.144; **Supplementary Table S5**).

### 3.3 Fusion-specific co-mutational architecture

RAS-pathway alterations were present in 17 of 32 CBFB::MYH11 cases (53.1%) and 27 of 138 RUNX1::RUNX1T1 cases (19.6%; odds ratio [OR], 4.66; 95% CI, 2.07–10.49; P < 0.001, q=0.002). The contrast was driven principally by NRAS, altered in 16 CBFB::MYH11 cases (50.0%) versus 21 RUNX1::RUNX1T1 cases (15.2%; OR, 5.57; 95% CI, 2.42–12.83; P < 0.001, q < 0.001). KRAS showed a nominal difference (18.8% vs 6.5%; P = 0.039) that did not withstand correction (q=0.256).

Conversely, chromatin-modifier alterations occurred in 34 RUNX1::RUNX1T1 cases (24.6%) and two CBFB::MYH11 cases (6.2%; P = 0.028, q=0.114). All observed ASXL1 (n=12) and ASXL2 (n=15) alterations occurred in RUNX1::RUNX1T1 AML, but neither gene-level comparison met the q < 0.05 threshold. Frequencies of KIT, FLT3, RTK alterations, signaling alterations overall, and at least two altered genes were not significantly different by fusion subtype (**Figure 2**; **Table 2**).

**Figure 2:**
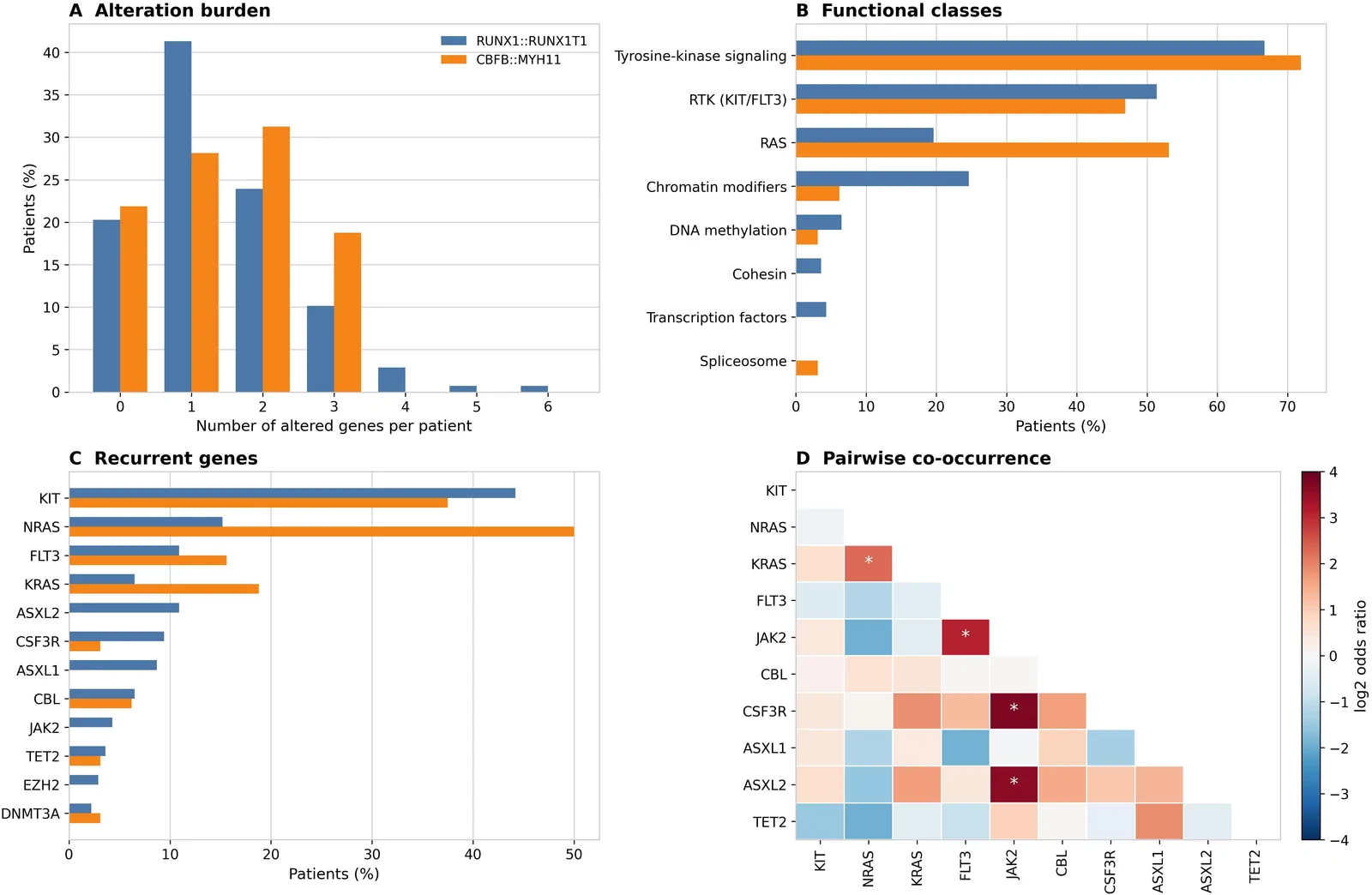
Fusion-specific genomic architecture. Panels show **(A)** number of altered genes per patient, **(B)** functional-category frequencies, **(C)** recurrent-gene frequencies, and **(D)** pairwise gene co-occurrence. Blue denotes RUNX1::RUNX1T1 and orange denotes CBFB::MYH11 AML. **(D)**, asterisks denote nominal P < 0.05; no pair remained significant after Benjamini–Hochberg correction. The robust fusion-specific difference after multiplicity correction was RAS-pathway, especially NRAS, enrichment in CBFB::MYH11 AML.

### 3.4 Associations with measurable residual disease

MFC-MRD was evaluable in 162 patients at TP1 and 156 at TP2; 58 (35.8%) and 29 (18.6%), respectively, were positive. TP2 molecular MRD data were evaluable in 156 patients, of whom 77 (49.4%) were positive. None of eight prespecified genomic features was associated with TP2 MFC-MRD or molecular MRD positivity after false-discovery-rate correction (MFC-MRD, all q≥0.836; molecular MRD, all q≥0.853; **Table 3**). Thus, the available data did not identify a diagnostic gene or pathway feature that reliably predicted post-induction MRD status.

**Table 3:** Associations of prespecified genomic features with MRD after induction 2.

Outcome	Feature	Present	Absent	OR (95% CI)	P	q
MFC-MRD	≥2 altered genes	11/63 (17.5%)	18/93 (19.4%)	0.89 (0.40–2.02)	0.836	0.836
MFC-MRD	Tyrosine-kinase signaling	21/108 (19.4%)	8/48 (16.7%)	1.17 (0.49–2.81)	0.824	0.836
MFC-MRD	RTK (KIT/FLT3)	16/80 (20.0%)	13/76 (17.1%)	1.20 (0.54–2.67)	0.685	0.836
MFC-MRD	RAS	6/42 (14.3%)	23/114 (20.2%)	0.69 (0.27–1.79)	0.491	0.836
MFC-MRD	KIT	14/69 (20.3%)	15/87 (17.2%)	1.22 (0.55–2.71)	0.681	0.836
MFC-MRD	FLT3 (any)	2/17 (11.8%)	27/139 (19.4%)	0.66 (0.16–2.67)	0.741	0.836
MFC-MRD	Chromatin modifiers	7/33 (21.2%)	22/123 (17.9%)	1.28 (0.50–3.24)	0.624	0.836
MFC-MRD	DNA methylation	2/9 (22.2%)	27/147 (18.4%)	1.46 (0.33–6.48)	0.674	0.836
Molecular MRD	≥2 altered genes	30/63 (47.6%)	47/93 (50.5%)	0.89 (0.47–1.68)	0.746	0.853
Molecular MRD	Tyrosine-kinase signaling	52/108 (48.1%)	25/48 (52.1%)	0.86 (0.44–1.68)	0.729	0.853
Molecular MRD	RTK (KIT/FLT3)	41/80 (51.2%)	36/76 (47.4%)	1.17 (0.62–2.18)	0.635	0.853
Molecular MRD	RAS	20/42 (47.6%)	57/114 (50.0%)	0.91 (0.45–1.84)	0.858	0.858
Molecular MRD	KIT	38/69 (55.1%)	39/87 (44.8%)	1.50 (0.80–2.82)	0.259	0.853
Molecular MRD	FLT3 (any)	5/17 (29.4%)	72/139 (51.8%)	0.41 (0.14–1.18)	0.122	0.853
Molecular MRD	Chromatin modifiers	15/33 (45.5%)	62/123 (50.4%)	0.82 (0.39–1.76)	0.696	0.853
Molecular MRD	DNA methylation	6/9 (66.7%)	71/147 (48.3%)	1.99 (0.52–7.58)	0.324	0.853

Present and absent columns show MRD-positive/evaluable n (%). OR > 1 indicates higher odds of MRD positivity when the genomic feature is present. q values are adjusted separately for MFC-MRD and molecular MRD comparisons.

### 3.5 Outcome and exploratory prognostic analyses

At a median follow-up of 33.2 months, estimated using the reverse Kaplan–Meier method, 25 EFS events and 14 deaths had occurred. EFS events comprised 11 relapses, eight treatment-related deaths, and six protocol-discontinuation events: three transfers after induction 1, one treatment change after induction 1 because of intolerance, one treatment abandonment after induction 1, and one transfer after induction 2. No induction failure occurred. Among patients achieving complete remission with evaluable relapse data, there were 11 relapses and six non-relapse deaths.

Three-year EFS was 84.2% (95% confidence interval [CI], 77.5%–89.1%), 3-year OS was 89.8% (95% CI, 83.2%–93.9%), and 3-year CIR was 7.7%. The numbers at risk at 12, 24, 36, and 48 months were 132, 95, 64, and 40 for EFS and 137, 101, 64, and 39 for OS. As shown in **Figure 3**, 3-year EFS was 84.0% in patients with <2 altered genes and 84.4% in those with ≥2 altered genes (log-rank P = 0.916), whereas 3-year OS was 91.4% and 87.6%, respectively (P = 0.470). For patients without versus with chromatin-modifier alterations, 3-year EFS was 82.6% versus 91.0% (P = 0.282), and 3-year OS was 88.2% versus 96.4% (P = 0.228). These unadjusted curves are descriptive; inference is based on the adjusted feature-specific models.

**Figure 3:**
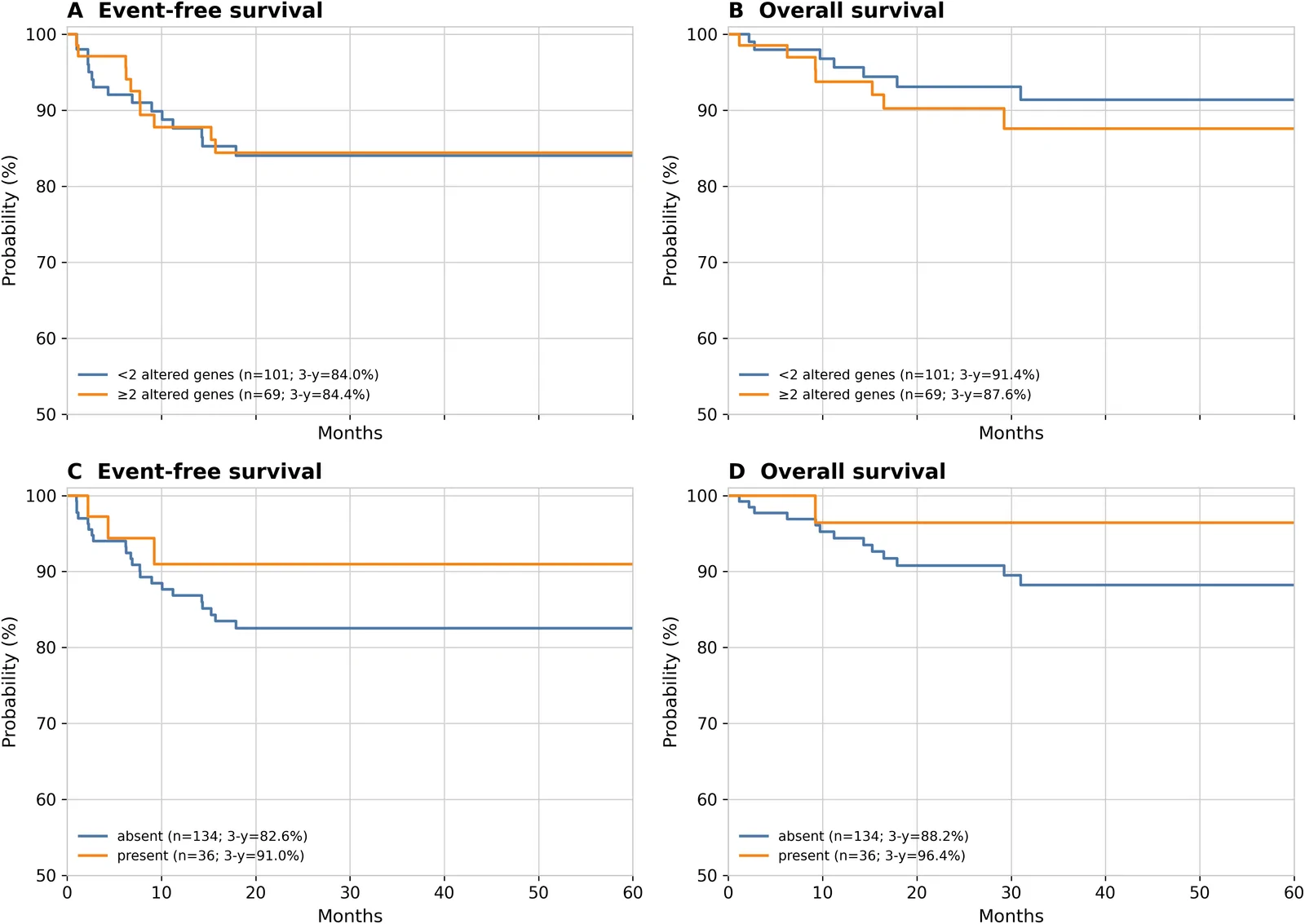
Descriptive Kaplan–Meier survival curves for broad genomic features. Panels show EFS **(A)** and OS **(B)** by alteration burden (<2 vs ≥2 altered genes), and EFS **(C)** and OS **(D)** by chromatin-modifier status. Curves are unadjusted and are provided for visualization; adjusted feature-specific estimates are reported in **Table 4** and **Supplementary Table S8**.

In separate Cox models adjusted for fusion subtype and induction regimen, no genomic feature was associated with EFS after false-discovery-rate correction. Estimates included at least two altered genes (hazard ratio [HR], 1.09; 95% CI, 0.48–2.50; P = 0.837), RAS alterations (HR, 0.69; 95% CI, 0.25–1.93; P = 0.480), KIT (HR, 0.64; 95% CI, 0.27–1.48; P = 0.295), FLT3 (HR, 1.76; 95% CI, 0.59–5.28; P = 0.314), and chromatin modifiers (HR, 0.63; 95% CI, 0.18–2.16; P = 0.461) (**Figure 4**; **Table 4**).

**Figure 4:**
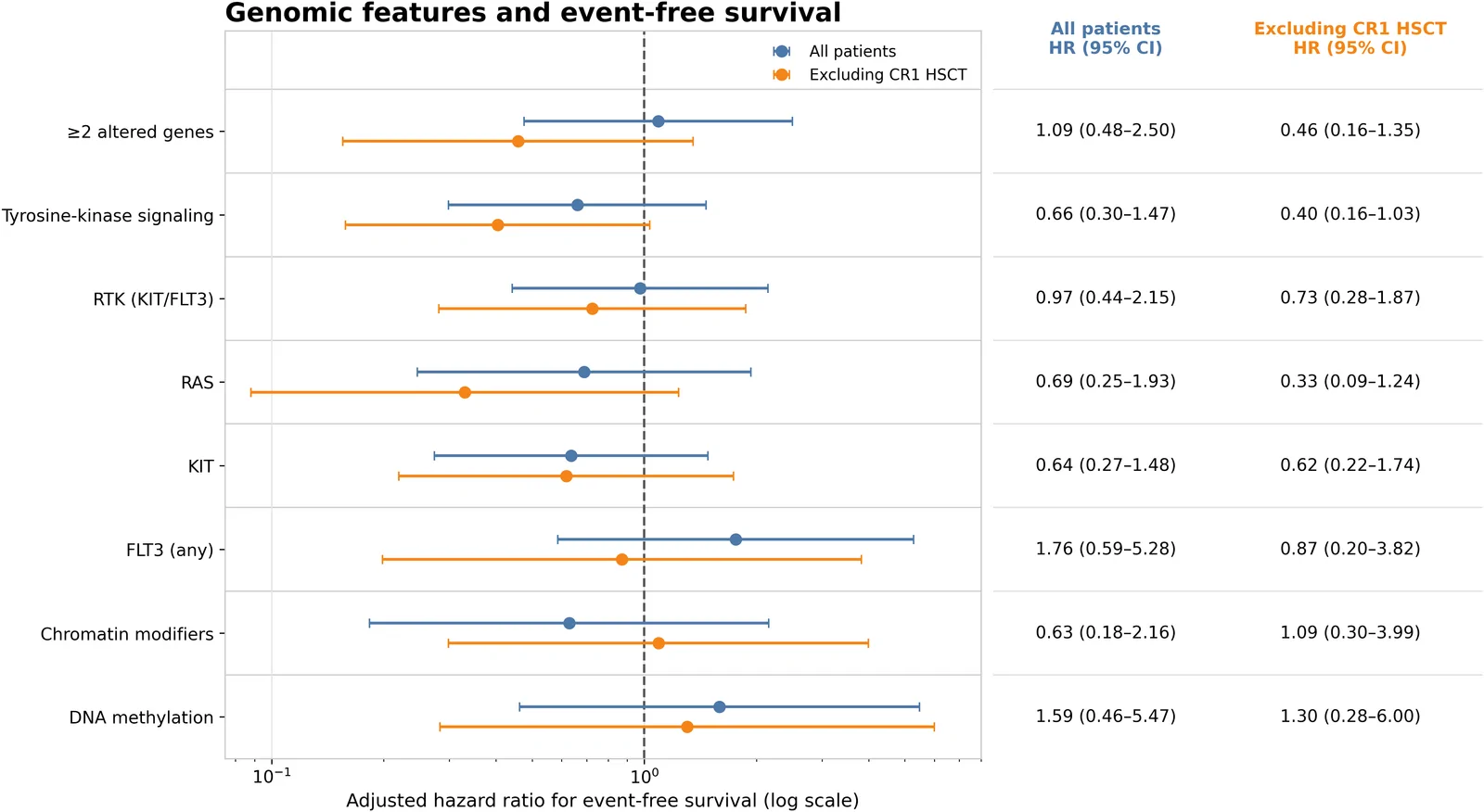
Adjusted associations of genomic features with event-free survival in the primary and sensitivity analyses. The forest plot shows adjusted hazard ratios and 95% confidence intervals from feature-specific Cox regression models. Blue circles show estimates from the primary analysis including all patients, whereas orange circles show estimates from the sensitivity analysis excluding patients who underwent hematopoietic stem cell transplantation in first complete remission. Both sets of estimates are displayed together for each genomic feature within a single forest plot; the models shown in this figure were adjusted only for fusion subtype and induction regimen. Expanded models additionally including age and presenting WBC count are reported separately in **Supplementary Table S9**.

**Table 4:** Feature-specific Cox models for event-free survival.

Feature	N	Events	Adjusted HR (95% CI)	P	q
≥2 altered genes	170	25	1.09 (0.48–2.50)	0.837	0.950
Tyrosine-kinase signaling	170	25	0.66 (0.30–1.47)	0.309	0.640
RTK (KIT/FLT3)	170	25	0.97 (0.44–2.15)	0.950	0.950
RAS	170	25	0.69 (0.25–1.93)	0.480	0.640
KIT	170	25	0.64 (0.27–1.48)	0.295	0.640
FLT3 (any)	170	25	1.76 (0.59–5.28)	0.314	0.640
Chromatin modifiers	170	25	0.63 (0.18–2.16)	0.461	0.640
DNA methylation	170	25	1.59 (0.46–5.47)	0.460	0.640

Each row represents a separate primary model adjusted only for fusion subtype and induction regimen. These estimates are not fully adjusted for all baseline prognostic factors. q values are Benjamini–Hochberg adjusted across the eight feature-specific EFS models; expanded models additionally including age and presenting WBC count are reported in **Supplementary Table S9**.

In the expanded complete-case baseline-covariate sensitivity analysis additionally adjusted for age and log-transformed presenting WBC count (n=163; 23 EFS events), estimates were broadly similar and no genomic feature was associated with EFS after false-discovery-rate correction (all q≥0.564; **Supplementary Table S9**). The smallest nominal P value was observed for KIT (HR, 0.44; 95% CI, 0.17–1.12; P = 0.083; q=0.564), but the confidence interval remained wide.

Results were materially unchanged after excluding 64 CR1-HSCT recipients (n=106; 19 EFS events; all q≥0.400; **Supplementary Table S6**) and in complete-case models additionally adjusted for TP2 MFC-MRD (n=156; 17 EFS events; all q≥0.770; **Supplementary Table S7**). Wide confidence intervals and sparse events preclude interpreting these null associations as proof of equivalence or absence of clinically important effects.

Adjusted OS and cause-specific relapse models were likewise imprecise and did not identify a significant genomic association after false-discovery-rate correction (**Supplementary Table S8**).

## 4 Discussion

In this WES-defined multicenter pediatric CBF-AML cohort, the cooperating genomic landscape was organized more clearly by fusion biology than by a single candidate gene. Signaling lesions were common in both entities, but RAS-pathway—and particularly NRAS—alterations were strongly enriched in CBFB::MYH11 AML. Chromatin-modifier alterations were observed predominantly in RUNX1::RUNX1T1 AML, although that comparison did not retain statistical significance after multiplicity correction. None of the prespecified baseline genomic features was established as a predictor of TP2 MRD or EFS in the available dataset.

The RAS-predominant architecture of CBFB::MYH11 AML accords with comprehensive CBF-AML sequencing studies and mechanistic models in which signaling lesions cooperate with the founding fusion (10–14, 20). Despite the small CBFB::MYH11 subgroup, the observed RAS-pathway and NRAS enrichments were large in magnitude (ORs, 4.66 and 5.57, respectively) and remained significant after false-discovery-rate correction. Conversely, the restriction of observed ASXL1/ASXL2 lesions to RUNX1::RUNX1T1 AML is directionally consistent with adult and mixed-age cohorts and with experimental evidence that ASXL2 loss cooperates with RUNX1::RUNX1T1 (10–13, 15). Nevertheless, the chromatin-modifier comparison had q=0.114, and the CBFB::MYH11 subgroup contained only 32 patients. The small subgroup particularly limits precision and power for less frequent lesions; therefore, nonsignificant fusion comparisons should not be interpreted as evidence that the subtypes are equivalent, and the observed architecture requires external confirmation.

The pediatric literature does not support a single, context-free prognostic interpretation of KIT, RAS, or FLT3 status. Reported associations vary by fusion subtype, functional class, allelic burden, co-mutation, treatment protocol, MRD response, and endpoint (16–22). In particular, functionally different KIT lesions may carry different effects (19), while clonal interference among signaling lesions can obscure binary gene-level associations (20). These considerations, together with only 25 EFS events, explain why the present models were deliberately feature-specific and parsimonious: each model contained one genomic feature and two prespecified adjustment variables, with no data-driven variable selection, genomic interaction, or multifeature prognostic model. The null estimates should narrow hypotheses for future validation, not close the question.

The absence of a multiplicity-adjusted association between diagnostic genomic features and TP2 MRD also warrants restraint. Prospective pediatric studies have established end-of-induction MFC-MRD as an important response marker (25), and international recommendations distinguish molecular and flow-based assays according to leukemia-specific targets and assay characteristics (26). Although MRD was assessed at TP1 and TP2, our genomic association analysis used protocol-recorded binary variables, primarily TP2 status, rather than continuous log reduction, standardized assay-level sensitivity, or a longitudinal model of serial kinetics. A relationship involving variant allele fraction, clone-specific clearance, or molecular-response kinetics could therefore remain undetected.

Tumor-only WES imposes a separate interpretive boundary. Population-frequency filtering, ClinVar/HGMD review, and expert curation reduce obvious artifacts but cannot prove somatic origin without paired normal material (31–34). We therefore excluded an explicitly germline KIT p.M541L call, removed WT1 records that conflated sequence and expression, and consistently used candidate leukemia-associated variant terminology. WES should also not be assumed to outperform deep targeted sequencing for low-allele-fraction variants, and the absence of source variant-call files or uniform depth metrics prevented an assay-sensitivity analysis.

The complete overlap with our previous clinical cohort is central to interpretation and is summarized explicitly in **Supplementary Table S11**. The 289-patient report evaluated clinically ascertained KIT and FLT3-ITD, MRD, induction regimen, and outcome (23). The present 170-patient analysis does not provide independent clinical validation; its incremental contribution is the broader conservatively curated WES co-mutational landscape, fusion-stratified and pathway-level comparisons with false-discovery-rate control, and exploratory genomic associations with response and outcome. Reuse of an openly disclosed cohort for a distinct genomic question does not make the observations independent, and we have not presented them as replication.

Additional limitations include retrospective design, selection based on WES availability, restriction to seven centers and a later diagnostic period than the parent cohort, a small CBFB::MYH11 subgroup, absence of matched germline DNA, limited characterization of copy-number and structural lesions, short follow-up for late relapse, and few outcome events relative to the number of candidate features. The selection audit was necessarily limited to three harmonizable characteristics; the higher clinical-source KIT positivity in the WES subset indicates residual selection and/or ascertainment differences, while unmeasured differences between included and non-included patients remain possible. The primary Cox models adjusted only for fusion subtype and induction regimen and therefore should not be interpreted as demonstrating independence from age, WBC count, or other baseline prognostic factors. Although the expanded sensitivity models additionally included age and WBC count, complete-case restriction left 23 EFS events across five model covariates, and the estimates remain vulnerable to overfitting, imprecision, and residual confounding. MRD was available as repeated binary measurements rather than standardized continuous kinetics. Treatment allocation and CR1-HSCT were selective; because transplant dates were unavailable, modeling eventual transplantation as a baseline exposure would risk immortal-time bias, and excluding recipients provides only a sensitivity analysis, not a treatment-effect estimate. FLT3 subtypes were too small for stable separate outcome estimates, and no patient received an FLT3 inhibitor. External validation with paired tumor-normal sequencing, standardized longitudinal MRD, larger fusion-specific strata, and prospectively captured treatment timing is required.

In conclusion, pediatric CBF-AML in this WES-defined cohort showed fusion-specific co-mutational architecture, with false-discovery-rate-adjusted enrichment of RAS-pathway and NRAS alterations in CBFB::MYH11 AML. No baseline gene or pathway feature was established as a predictor of TP2 MRD or EFS in the prespecified models. These retrospective findings are descriptive and hypothesis-generating; they support biologically stratified validation studies but not mutation-directed treatment change, regimen-specific causal inference, or generalization to all patients in the parent clinical cohort.

## Data Availability

The raw data supporting the conclusions of this article will be made available by the authors, without undue reservation.
