# Supplementary material for: Fusion-specific co-mutational architecture and treatment-response associations in pediatric core-binding factor acute myeloid leukemia: a multicenter exome-profiled cohort
Source: Front Oncol. 2026 Sep 30;16:1897872. doi: 10.3389/fonc.2026.1897872 (PMC13661505; doi:10.3389/fonc.2026.1897872)
Supplement: Supplementary file 1 [file SupplementaryFile1.docx]

**Supplementary material**

**Fusion-specific co-mutational architecture and treatment-response associations in pediatric core-binding factor acute myeloid leukemia**

*Mei Li, Chunxia Cai, Chunping Wu, Shaohua Le, Nainong Li, and Yongzhi Zheng*

# Contents

| **Item** | **Description** |
| --- | --- |
| Supplementary Figure S1 | C-HUANAN-AML-15 treatment protocol and regimen details |
| Supplementary Figure S2 | Selection of the WES-defined cohort and treatment disposition |
| Supplementary Table S1 | Genomic data-curation rules used in the primary analysis |
| Supplementary Table S2 | Prespecified functional pathway membership |
| Supplementary Table S3 | Curated KIT variant distribution |
| Supplementary Table S4 | Patient-anonymized FLT3 source categories and recorded annotations |
| Supplementary Table S5 | Pairwise co-occurrence among recurrently altered genes |
| Supplementary Table S6 | EFS Cox sensitivity analysis excluding CR1-HSCT recipients |
| Supplementary Table S7 | EFS Cox sensitivity analysis adjusted for TP2 MFC-MRD |
| Supplementary Table S8 | Adjusted OS and relapse cause-specific models |
| Supplementary Table S9 | EFS Cox sensitivity analysis additionally adjusted for age and presenting WBC count |
| Supplementary Table S10 | Limited comparison of WES-included and non-included patients |
| Supplementary Table S11 | Relationship between the prior clinical report and the current WES analysis |

# Supplementary methods

## Multiplicity control

Benjamini–Hochberg adjustment was applied within conceptually related analysis families. Fusion comparisons were adjusted separately for summary features, pathways, and genes/subtypes. MRD comparisons were adjusted separately for TP2 MFC-MRD and TP2 molecular MRD. Survival q values were adjusted within each endpoint and sensitivity-analysis family. Pairwise co-occurrence q values were adjusted across all tested gene pairs.

## Handling of transplantation

The database recorded whether HSCT occurred and remission status before HSCT but did not provide a uniform transplant date suitable for time-dependent covariate modeling. Treating eventual CR1-HSCT as a baseline covariate would create immortal-time bias. The primary models therefore followed all patients from diagnosis and did not adjust for future transplantation. A prespecified sensitivity analysis excluded CR1-HSCT recipients.

## Handling of post-induction MRD

TP2 MFC-MRD is a post-baseline treatment-response variable and may mediate, rather than confound, associations between baseline genomics and EFS. It was therefore excluded from primary baseline models and included in a secondary complete-case sensitivity analysis only. Results should not be interpreted as causal mediation estimates.

## Expanded baseline-covariate sensitivity analysis

To assess whether the primary genomic-feature estimates were materially altered by baseline age and leukocytosis, each feature-specific Cox model was expanded to additionally include age at diagnosis (continuous, years) and presenting WBC count [log-transformed as log(1+WBC), with WBC measured in ×10⁹/L], together with fusion subtype and induction regimen. Analyses used complete cases (n=163; 23 EFS events). Given the small number of events relative to five covariates in each model, these estimates are exploratory and do not establish independence from all baseline prognostic factors.

## Selection-bias audit

The 170 WES-included patients were compared with the 119 patients in the previously reported 289-patient CBF-AML cohort who were not included in the current analysis. The audit was limited to fusion subtype, clinically ascertained KIT positivity, and FLT3-ITD because these characteristics could be reconstructed under comparable definitions from the current analytic file and the published full-cohort counts. Non-included counts were calculated by subtraction. Fisher's exact tests and absolute standardized differences were descriptive. The earlier clinical-source KIT variable was generated by PCR before 2019 and WES thereafter and therefore differs from the conservative candidate-variant definition used in the current WES matrix.

# Supplementary Figure S1. C-HUANAN-AML-15 treatment protocol

Note. The diagram summarizes diagnostic bone-marrow assessment, nonrandom allocation to FLAG-IDA or DAE induction, response-adapted referral for HSCT, and consolidation with HAE/HHA followed by MidAC. Children aged <1 year received a 25% dose reduction. BM, bone marrow; CR, complete remission; HSCT, hematopoietic stem-cell transplantation.

# Supplementary Figure S2. Selection of the WES-defined cohort and treatment disposition

Note. Among 875 children with newly diagnosed acute myeloid leukemia other than acute promyelocytic leukemia who were treated according to the C-HUANAN-AML-15 protocol, 289 had CBF-AML. The present analysis included 170 patients treated at seven participating centers between 2019 and 2023 for whom diagnostic WES data were available. Allocation to FLAG-IDA or DAE induction was nonrandom. The diagram summarizes induction treatment, response assessment, discontinuation or transfer, treatment-related mortality, HSCT, and progression through consolidation therapy. Treatment status after consolidation is shown only for patients remaining on the corresponding treatment pathway and does not represent the cumulative distribution of all EFS events during follow-up. CBF-AML, core-binding factor acute myeloid leukemia; CCR, continuous complete remission; CR, complete remission; DAE, daunorubicin, cytarabine, and etoposide; FLAG-IDA, fludarabine, cytarabine, granulocyte colony-stimulating factor, and idarubicin; HSCT, hematopoietic stem-cell transplantation; TRM, treatment-related mortality; WES, whole-exome sequencing.

**Supplementary Table S1. Genomic data-curation rules used in the primary analysis**

| **Item** | **Source issue/rule** | **Primary-analysis decision** |
| --- | --- | --- |
| Somatic status | Matched normal unavailable | Use ‘candidate leukemia-associated variant’; do not claim confirmed somatic origin |
| Population filtering | MAF >1% in population/in-house databases | Exclude common polymorphisms |
| KIT p.M541L | Explicitly labeled germline | Exclude M541L; retain another pathogenic KIT call in the same patient |
| WT1 | Mutation flag mixed with high-expression/transcript annotations | Exclude all 15 WT1 source flags from primary mutation matrix |
| FLT3 non-ITD | All non-ITD records classified as other FLT3 mutations | Describe ITD and other non-ITD categories; do not perform subtype-specific survival inference |
| Mutation count | Number of curated genes | Count patient–gene events, not individual variants |
| Pathway positivity | At least one altered member gene | Binary pathway feature per patient |
| Co-occurrence | Genes altered in ≥5 patients | Fisher’s exact test with FDR correction |

**Supplementary Table S2. Prespecified functional pathway membership**

| **Functional category** | **Member genes** |
| --- | --- |
| Tyrosine-kinase signaling | KIT, FLT3, NRAS, KRAS, JAK2, PTPN11, CBL, CSF3R |
| RTK | KIT, FLT3 |
| RAS | NRAS, KRAS |
| Chromatin modifiers | ASXL1, ASXL2, KDM6A, EZH2, BCORL1, KMT2A |
| DNA methylation | TET2, DNMT3A, IDH2 |
| Cohesin | RAD21, SMC1A, SMC3, STAG2 |
| Transcription factors | ETV6, RUNX1, IKZF1 |
| Spliceosome | SRSF2 |

*Note. A pathway was coded positive when at least one listed gene carried a curated candidate leukemia-associated variant.*

**Supplementary Table S3. Curated KIT variant distribution**

| **Recorded exon** | **Normalized recorded variant** | **Variant records, n** |
| --- | --- | --- |
| 11 | V559A | 1 |
| 11 | Y570delinsYIDPTQLPYDHKWEFH | 1 |
| 11 | in-frame delins (site not specified) | 1 |
| 17 | N822K | 33 |
| 17 | D816Y | 12 |
| 17 | D816V | 8 |
| 17 | D816H | 5 |
| 17 | D820G | 4 |
| 17 | D820Y | 3 |
| 17 | D816 (substitution not specified) | 1 |
| 8 | D419del | 6 |
| 8 | Y418_D419 indel | 3 |
| 8 | T417_D419delinsV | 2 |
| 8 | L416_D419delinsLL | 1 |
| 8 | T417_D419del | 1 |
| 8 | T417_D419delinsL | 1 |
| 8 | Y418/D419-region indel | 1 |
| 8 | Y418_D419insFL | 1 |
| 9 | in-frame insertion (site not specified) | 2 |
| Unspecified | c.1249 1256delinsGT | 1 |

*Note. The table counts recorded variant calls, not patients. KIT p.M541L calls explicitly labeled germline were excluded. Source spelling was normalized only for readability; original VCF-level verification was unavailable.*

**Supplementary Table S4. Patient-anonymized FLT3 source categories and recorded annotations**

| **Study case** | **Fusion** | **Source category** | **Recorded FLT3 annotation** |
| --- | --- | --- | --- |
| Case F01 | RUNX1::RUNX1T1 | Other non-ITD | FLT3:NM_004119:exon14:c.T1775C:p.V592A |
| Case F02 | RUNX1::RUNX1T1 | Other non-ITD | FLT3-positive; variant annotation not specified in source dataset |
| Case F03 | CBFB::MYH11 | Other non-ITD | FLT3:NM_004119:exon20:c.2503_2505del:p.835_835del ; FLT3:NM_004119:exon16:c.C2039T:p.A680V |
| Case F04 | RUNX1::RUNX1T1 | Other non-ITD | FLT3high(p.K602delinsRPRYVDFREYEYD LK) 53.0% |
| Case F05 | CBFB::MYH11 | Other non-ITD | FLT3: p.D835Y 1.1% |
| Case F06 | RUNX1::RUNX1T1 | Other non-ITD | FLT3:p.D835Y 43.0% |
| Case F07 | RUNX1::RUNX1T1 | Other non-ITD | FLT3-TKD(p.D835H) |
| Case F08 | RUNX1::RUNX1T1 | ITD | FLT3-positive; variant annotation not specified in source dataset |
| Case F09 | CBFB::MYH11 | Other non-ITD | FLT3(d835y、N676K) KIT、FLT3 |
| Case F10 | RUNX1::RUNX1T1 | Other non-ITD | FLT3-TKD KIT、ASXL1、FLT3-TKD |
| Case F11 | CBFB::MYH11 | Other non-ITD | FLT3-positive; variant annotation not specified in source dataset |
| Case F12 | RUNX1::RUNX1T1 | ITD | FLT3-ITD |
| Case F13 | RUNX1::RUNX1T1 | ITD | FLT3-ITD |
| Case F14 | RUNX1::RUNX1T1 | Other non-ITD | FLT3-TKD |
| Case F15 | RUNX1::RUNX1T1 | ITD | FLT3-positive; variant annotation not specified in source dataset |
| Case F16 | RUNX1::RUNX1T1 | Other non-ITD | FLT3-positive; variant annotation not specified in source dataset |
| Case F17 | CBFB::MYH11 | ITD | FLT3-ITD |
| Case F18 | RUNX1::RUNX1T1 | ITD | FLT3-ITD |
| Case F19 | RUNX1::RUNX1T1 | ITD | FLT3-ITD |
| Case F20 | RUNX1::RUNX1T1 | ITD | FLT3-ITD |

*Note. Study-case labels are generated for this table and contain no patient identifiers. The verified classification comprised 8 FLT3-ITD records and 12 other non-ITD FLT3 records. All non-ITD alterations were grouped as other FLT3 mutations; no separate TKD outcome analysis was performed.*

**Supplementary Table S5. Pairwise co-occurrence among recurrently altered genes**

| **Gene 1** | **Gene 2** | **Both** | **OR (95% CI)** | **P** | **q** |
| --- | --- | --- | --- | --- | --- |
| NRAS | KRAS | 8 | 4.86 (1.68–14.04) | 0.005 | 0.144 |
| JAK2 | CSF3R | 3 | 13.35 (2.70–65.99) | 0.008 | 0.144 |
| JAK2 | ASXL2 | 3 | 12.20 (2.49–59.82) | 0.01 | 0.144 |
| FLT3 | JAK2 | 3 | 8.43 (1.77–40.20) | 0.022 | 0.251 |
| KRAS | CSF3R | 3 | 3.52 (0.93–13.29) | 0.112 | 0.91 |
| KRAS | ASXL2 | 3 | 3.21 (0.86–12.02) | 0.132 | 0.91 |
| NRAS | ASXL2 | 1 | 0.34 (0.06–1.89) | 0.196 | 0.91 |
| FLT3 | CSF3R | 3 | 2.43 (0.66–8.86) | 0.217 | 0.91 |
| CBL | CSF3R | 2 | 3.11 (0.69–14.03) | 0.226 | 0.91 |
| KIT | TET2 | 1 | 0.35 (0.06–2.17) | 0.239 | 0.91 |
| CBL | ASXL2 | 2 | 2.86 (0.64–12.81) | 0.251 | 0.91 |
| NRAS | FLT3 | 2 | 0.44 (0.11–1.74) | 0.251 | 0.91 |
| ASXL1 | ASXL2 | 2 | 2.57 (0.58–11.36) | 0.286 | 0.91 |
| NRAS | JAK2 | 0 | 0.26 (0.01–4.75) | 0.342 | 0.91 |
| NRAS | TET2 | 0 | 0.26 (0.01–4.75) | 0.342 | 0.91 |
| CSF3R | ASXL2 | 2 | 2.13 (0.49–9.23) | 0.356 | 0.91 |
| ASXL1 | TET2 | 1 | 3.64 (0.54–24.39) | 0.36 | 0.91 |
| FLT3 | ASXL1 | 0 | 0.27 (0.02–4.74) | 0.364 | 0.91 |
| KIT | ASXL2 | 8 | 1.57 (0.56–4.40) | 0.424 | 0.954 |
| KIT | KRAS | 8 | 1.57 (0.56–4.40) | 0.424 | 0.954 |
| NRAS | ASXL1 | 1 | 0.44 (0.08–2.50) | 0.466 | 0.986 |
| KIT | FLT3 | 7 | 0.71 (0.27–1.82) | 0.482 | 0.986 |
| CBL | ASXL1 | 1 | 1.84 (0.30–11.31) | 0.564 | 1.0 |
| KIT | CSF3R | 7 | 1.36 (0.47–3.93) | 0.587 | 1.0 |
| CSF3R | ASXL1 | 0 | 0.40 (0.02–7.09) | 0.602 | 1.0 |
| FLT3 | ASXL2 | 2 | 1.38 (0.33–5.78) | 0.691 | 1.0 |
| NRAS | CBL | 3 | 1.50 (0.41–5.50) | 0.706 | 1.0 |
| KIT | ASXL1 | 6 | 1.36 (0.44–4.20) | 0.764 | 1.0 |
| KIT | NRAS | 15 | 0.89 (0.43–1.85) | 0.852 | 1.0 |
| ASXL2 | TET2 | 0 | 0.74 (0.04–13.81) | 1.0 | 1.0 |
| CBL | TET2 | 0 | 1.03 (0.05–19.39) | 1.0 | 1.0 |
| CSF3R | TET2 | 0 | 0.80 (0.04–14.90) | 1.0 | 1.0 |
| FLT3 | CBL | 1 | 1.03 (0.17–6.07) | 1.0 | 1.0 |
| FLT3 | TET2 | 0 | 0.54 (0.03–9.99) | 1.0 | 1.0 |
| JAK2 | ASXL1 | 0 | 0.94 (0.05–17.64) | 1.0 | 1.0 |
| JAK2 | CBL | 0 | 1.03 (0.05–19.39) | 1.0 | 1.0 |
| JAK2 | TET2 | 0 | 1.88 (0.10–36.99) | 1.0 | 1.0 |
| KIT | CBL | 5 | 1.13 (0.35–3.67) | 1.0 | 1.0 |
| KIT | JAK2 | 3 | 1.34 (0.30–6.09) | 1.0 | 1.0 |
| KRAS | ASXL1 | 1 | 1.30 (0.22–7.75) | 1.0 | 1.0 |
| KRAS | CBL | 1 | 1.43 (0.24–8.62) | 1.0 | 1.0 |
| KRAS | FLT3 | 1 | 0.72 (0.13–4.15) | 1.0 | 1.0 |
| KRAS | JAK2 | 0 | 0.74 (0.04–13.81) | 1.0 | 1.0 |
| KRAS | TET2 | 0 | 0.74 (0.04–13.81) | 1.0 | 1.0 |
| NRAS | CSF3R | 3 | 1.08 (0.31–3.79) | 1.0 | 1.0 |

*Note. Only genes altered in at least five patients were tested. No pair remained significant at q<0.05.*

**Supplementary Table S6. EFS Cox sensitivity analysis excluding CR1-HSCT recipients**

| **Feature** | **N** | **Events** | **Adjusted HR (95% CI)** | **P** | **q** |
| --- | --- | --- | --- | --- | --- |
| ≥2 altered genes | 106 | 19 | 0.46 (0.16–1.35) | 0.158 | 0.421 |
| Tyrosine-kinase signaling | 106 | 19 | 0.40 (0.16–1.03) | 0.059 | 0.400 |
| RTK (KIT/FLT3) | 106 | 19 | 0.73 (0.28–1.87) | 0.507 | 0.812 |
| RAS | 106 | 19 | 0.33 (0.09–1.24) | 0.100 | 0.400 |
| KIT | 106 | 19 | 0.62 (0.22–1.74) | 0.361 | 0.721 |
| FLT3 (any) | 106 | 19 | 0.87 (0.20–3.82) | 0.855 | 0.894 |
| Chromatin modifiers | 106 | 19 | 1.09 (0.30–3.99) | 0.894 | 0.894 |
| DNA methylation | 106 | 19 | 1.30 (0.28–6.00) | 0.733 | 0.894 |

*Note. Each feature was evaluated in a separate model adjusted for fusion subtype and induction regimen. Sixty-four CR1-HSCT recipients were excluded.*

**Supplementary Table S7. EFS Cox sensitivity analysis adjusted for TP2 MFC-MRD**

| **Feature** | **N** | **Events** | **Adjusted HR (95% CI)** | **P** | **q** |
| --- | --- | --- | --- | --- | --- |
| ≥2 altered genes | 156 | 17 | 1.52 (0.56–4.17) | 0.415 | 0.770 |
| Tyrosine-kinase signaling | 156 | 17 | 0.57 (0.22–1.52) | 0.263 | 0.770 |
| RTK (KIT/FLT3) | 156 | 17 | 0.73 (0.27–1.96) | 0.538 | 0.770 |
| RAS | 156 | 17 | 0.84 (0.25–2.76) | 0.770 | 0.770 |
| KIT | 156 | 17 | 0.55 (0.19–1.58) | 0.264 | 0.770 |
| FLT3 (any) | 156 | 17 | 1.48 (0.32–6.84) | 0.618 | 0.770 |
| Chromatin modifiers | 156 | 17 | 0.73 (0.16–3.41) | 0.693 | 0.770 |
| DNA methylation | 156 | 17 | 1.90 (0.50–7.26) | 0.350 | 0.770 |

*Note. Complete-case models adjusted for fusion subtype, induction regimen, and TP2 MFC-MRD. MRD is post-baseline; these models are secondary and non-causal.*

**Supplementary Table S8. Adjusted OS and relapse cause-specific models**

| **Endpoint** | **Feature** | **N** | **Events** | **Adjusted HR (95% CI)** | **P** | **q** |
| --- | --- | --- | --- | --- | --- | --- |
| OS | ≥2 altered genes | 170 | 14 | 1.9 (0.63–5.7) | 0.259 | 0.664 |
| OS | Tyrosine-kinase signaling | 170 | 14 | 0.78 (0.26–2.3) | 0.644 | 0.736 |
| OS | RTK (KIT/FLT3) | 170 | 14 | 0.84 (0.29–2.4) | 0.745 | 0.745 |
| OS | RAS | 170 | 14 | 1.8 (0.56–5.6) | 0.332 | 0.664 |
| OS | KIT | 170 | 14 | 0.56 (0.17–1.8) | 0.33 | 0.664 |
| OS | FLT3 (any) | 170 | 14 | 1.7 (0.35–7.9) | 0.514 | 0.686 |
| OS | Chromatin modifiers | 170 | 14 | 0.4 (0.051–3.2) | 0.386 | 0.664 |
| OS | DNA methylation | 170 | 14 | 1.9 (0.41–8.8) | 0.415 | 0.664 |
| Relapse cause-specific | ≥2 altered genes | 163 | 11 | 0.99 (0.28–3.6) | 0.993 | 0.993 |
| Relapse cause-specific | Tyrosine-kinase signaling | 163 | 11 | 0.34 (0.097–1.2) | 0.088 | 0.707 |
| Relapse cause-specific | RTK (KIT/FLT3) | 163 | 11 | 0.44 (0.12–1.7) | 0.23 | 0.744 |
| Relapse cause-specific | RAS | 163 | 11 | 0.55 (0.11–2.7) | 0.465 | 0.744 |
| Relapse cause-specific | KIT | 163 | 11 | 0.55 (0.14–2.1) | 0.378 | 0.744 |
| Relapse cause-specific | FLT3 (any) | 163 | 11 | 4.5e-05 (1.1e-128–1.9e+119) | 0.945 | 0.993 |
| Relapse cause-specific | Chromatin modifiers | 163 | 11 | 4.5e-05 (9.4e-101–2.2e+91) | 0.929 | 0.993 |
| Relapse cause-specific | DNA methylation | 163 | 11 | 2 (0.41–10) | 0.388 | 0.744 |

*Note. Each row is a separate model adjusted for fusion subtype and induction regimen. Extreme or very wide relapse-model intervals reflect sparse events and quasi-separation; these estimates are not clinically interpretable.*

# Supplementary Table S9. EFS Cox sensitivity analysis additionally adjusted for age and presenting WBC count

| **Feature** | **N** | **Events** | **Adjusted HR (95% CI)** | **P** | **q** |
| --- | --- | --- | --- | --- | --- |
| ≥2 altered genes | 163 | 23 | 1.15 (0.48–2.76) | 0.762 | 0.859 |
| Tyrosine-kinase signaling | 163 | 23 | 0.54 (0.24–1.23) | 0.141 | 0.564 |
| RTK (KIT/FLT3) | 163 | 23 | 0.74 (0.32–1.71) | 0.486 | 0.765 |
| RAS | 163 | 23 | 0.91 (0.32–2.56) | 0.859 | 0.859 |
| KIT | 163 | 23 | 0.44 (0.17–1.12) | 0.083 | 0.564 |
| FLT3 (any) | 163 | 23 | 2.02 (0.63–6.43) | 0.234 | 0.624 |
| Chromatin modifiers | 163 | 23 | 0.70 (0.20–2.45) | 0.573 | 0.765 |
| DNA methylation | 163 | 23 | 1.88 (0.55–6.46) | 0.316 | 0.633 |

Note. Each row represents a separate complete-case Cox model including the genomic feature, fusion subtype, induction regimen, age at diagnosis (continuous, years), and presenting WBC count [log-transformed as log(1+WBC), with WBC measured in ×10⁹/L]. Age and/or WBC data were unavailable for seven patients; the analysis therefore included 163 patients and 23 EFS events. P values are two-sided Wald tests; q values are Benjamini–Hochberg adjusted across the eight feature-specific models. Because of the limited event count, these models are exploratory and should not be interpreted as fully adjusted definitive estimates. CI, confidence interval; EFS, event-free survival; HR, hazard ratio; WBC, white blood cell count.

# Supplementary Table S10. Limited comparison of WES-included and non-included patients from the previously reported clinical cohort

| **Characteristic** | **WES included (n=170)** | **Not included (n=119)** | **Absolute standardized difference** | **Fisher's exact P** |
| --- | --- | --- | --- | --- |
| RUNX1::RUNX1T1 | 138 (81.2%) | 93 (78.2%) | 0.075 | 0.553 |
| KIT positive, prior clinical-source definition | 74 (43.5%) | 29 (24.4%) | 0.413 | 0.001 |
| FLT3-ITD positive | 8 (4.7%) | 8 (6.7%) | 0.087 | 0.602 |

Note. All 170 WES-included patients were part of the previously reported 289-patient C-HUANAN-AML-15 CBF-AML cohort; the non-included group therefore comprised 119 patients. Non-included counts were derived by subtracting current-subset counts from published full-cohort counts. The comparison was limited to variables recoverable under harmonized definitions; individual-level comparison across the full baseline dataset was not possible. The prior clinical-source KIT definition reflects PCR screening before 2019 and WES thereafter and is not identical to the conservative candidate leukemia-associated variant definition used in the current genomic matrix (73 curated KIT-positive patients after exclusion of a p.M541L-only call). Absolute standardized differences are reported as magnitudes. P values are descriptive and were not adjusted for multiplicity. ITD, internal tandem duplication; WES, whole-exome sequencing.

# Supplementary Table S11. Relationship between the prior clinical report and the current WES-defined analysis

| **Dimension** | **Prior clinical report** | **Current WES-defined analysis** | **Interpretive implication** |
| --- | --- | --- | --- |
| Population | 289 children with CBF-AML; 11 centers; 2015–2023 | 170 of the same children; seven centers; 2019–2023; diagnostic WES available | Complete overlap of the current cohort; not an independent validation cohort |
| Molecular scope | Clinically ascertained KIT and FLT3-ITD | Conservatively curated candidate variants across genes and prespecified functional pathways | Broader genomic scope, with tumor-only limitations |
| Primary question | Clinical associations of KIT/FLT3-ITD, MRD, regimen, and outcome | Fusion-specific co-mutational architecture and exploratory genomic associations with TP2 MRD and outcome | Distinct analytic question using a subset of the same clinical cohort |
| Multiplicity and modeling | Candidate-gene clinical analyses | Prespecified comparison families with Benjamini–Hochberg control; one genomic feature per parsimonious Cox model | Current results should be interpreted as descriptive and hypothesis-generating |
| Claims not made | — | No independent replication, germline inference, treatment-effect comparison, or mutation-specific causal claim | Defines the boundary of novelty and inference |

Note. The prior clinical report is Zheng et al. (reference 23 in the main manuscript). The present analysis reuses the WES-defined subset for a distinct genomic question and does not represent external or internal independent replication. CBF-AML, core-binding factor acute myeloid leukemia; MRD, measurable residual disease; TP2, after the second induction course; WES, whole-exome sequencing.
